# Prenatal Exposure to Carbamazepine Reduces Hippocampal and Cortical Neuronal Cell Population in New-Born and Young Mice without Detectable Effects on Learning And Memory

**DOI:** 10.1371/journal.pone.0080497

**Published:** 2013-11-14

**Authors:** Elin Åberg, Sarah Holst, Alexandru Neagu, Sven Ove Ögren, Catharina Lavebratt

**Affiliations:** 1 Neurogenetics Unit, Department of Molecular Medicine and Surgery, Karolinska Institutet, Stockholm, Sweden and Center for Molecular Medicine, Karolinska University Hospital, Stockholm, Sweden; 2 Department of Neuroscience, Karolinska Institute, Stockholm, Sweden; Fudan University, China

## Abstract

Pregnant women with epilepsy have to balance maternal and fetal risks associated with uncontrolled seizures against the potential teratogenic effects from antiepileptic drugs (AEDs). Carbamazepine (CBZ) is among the four most commonly used AEDs for treatment of pregnant epileptic women. We previously reported that new-born children had a decreased head circumference after *in utero* CBZ exposure. This study investigates how prenatal exposure of CBZ influences the number of neurons in new-born and young mouse hippocampus, amygdala and cortex cerebri. Clinical studies describe inconclusive results on if prenatal CBZ treatment influences cognition. Here we investigate this issue in mice using two well characterized cognitive tasks, the passive avoidance test and the Morris water maze test. Prenatal exposure of CBZ reduced the number of neurons (NeuN-immunoreactive cells) in the new-born mouse hippocampus with 50% compared to non-exposed mice. A reduction of neurons (20%) in hippocampus was still observed when the animals were 5 weeks old. These mice also displayed a 25% reduction of neurons in cortex cerebri. Prenatal CBZ treatment did not significantly impair learning and memory measured in the passive avoidance test and in the Morris water maze. However, these mice displayed a higher degree of thigmotaxic behaviour than the control mice. The body weight of prenatally CBZ exposed five-week old mice were lower compared to control mice not exposed to CBZ (p = 0.001). In conclusion, prenatal exposure to CBZ reduces the number of neurons dramatically in areas important for cognition such as hippocampus and cortex, without severe impairments on learning and memory. These results are in line with some clinical studies, reporting that CBZ has minor negative effects on cognition. The challenge for future studies are to segment out what possible effects a reduction of neurons could have on different types of cognition, like intellectual ability and social interaction.

## Introduction

Epilepsy affects 1–2% of humans worldwide and one third of those are women in reproductive age [1], [2]. Both seizures and antiepileptic drug (AED) treatment during pregnancy are thought to influence the child negatively. The risk of major fetal congenital malformations as a cause of the disease is approximately 1–2% [3], [4] but this risk is even 2–3 fold higher in women with epilepsy treated with AEDs [5], [6], [7], [8]. Thus, pregnant women with epilepsy have to balance maternal and fetal risks associated with uncontrolled seizures against the potential teratogenic effects from AEDs. The most commonly used AEDs for treatment of pregnant epileptic women today are valproate (VPA), phenytoin (PHT), carbamazepine (CBZ) and lamotrigine (LTG) [9].

AEDs are not only subscribed to individuals with epilepsy, but are also used as mood stabilizers in psychiatric disorders [10]. In addition, pregnancy increases the risk for the onset and recurrence of several psychiatric disorders and pregnant women suffering from affective disorders do often require treatment with mood stabilizers (lithium carbonate, VPA, CBZ, LTG), antidepressants and/or antipsychotics [11], [12].

Congenital heart disease, cleft lip and/or palate, limb defects, genitourinary malformations and neural tube defects are malformations seen after AED treatment [13], [14], [15], [16]. Also cognitive impairments, intellectual disability and maladaptive behaviors are associated with exposure to AEDs *in utero* [9], [17], [18], [19], [20], [21]. It is widely accepted that VPA is associated with more side effects compared to the other commonly used AEDs and that polytherapy regimes are more harmful than monotherapy regimes [9], [17], [18], [19], [21], [22], [23], [24], [25], [26], [27], [28], [29]. Long-term effects into adulthood induced by *in utero* exposure of AEDs have not been extensively studied, even though there are some follow up studies indicating that almost all AEDs have negative effects on later intellectual functioning [20], [30], [31], [32], [33]. Moreover, the mechanisms behind cognitive impairments caused by AEDs are still not well described although a few animal studies have given some clues [34], [35], [36].

We made a retrospective study on all Swedish singletons births between 1995 and 2005 (over 900.000 births) obtained from the Swedish medical birth registry, and demonstrated a reduced head circumference at birth in newborns exposed to CBZ *in utero* [37]. Previous studies of cognitive performances in young children (up till 6 years of age) exposed to CBZ *in utero* demonstrate conflicting results. Some investigations demonstrate general developmental delay, impaired language and IQ scores [38], [39], [40], [41], while other studies could not detect any cognitive difference in performances between CBZ exposed children and controls [20], [29], [42], [43].

The present study was conducted to investigate whether *in utero* exposure to CBZ can affect the neuronal hippocampal cell population in newborn mice. In addition, we examined the effect of prenatal CBZ exposure on neuronal cell populations in hippocampus, cortex cerebri and amygdala in five-week old mice and related these changes to cognitive performances. Cognitive performance was evaluated by use of the Passive Avoidance (PA) test and the Morris water maze test, which are well characterised cognitive tasks of emotional [44], [45] respective spatial learning and memory. Two different mouse strains (BALB/c mice and C57BL/6 mice) were used in this study. The BALB/c mice were used in the PA test and for histological analysis, but since they were poorly performers in the Morris water maze test, we used C57BL/6 mice for this task.

## Materials and Methods

### Ethics Statement

All animals were handled according to guidelines approved by a local ethics committee (Stockholm Northern Ethics Board of Animal Experimentation) in Sweden.

### Animals

Male and female mice, BALB/c and C57BL/6 (Scanbur, Sweden; Taconics, Germany) were used to produce offspring examined in this study. They were housed, 2–7/cage (offspring) or 2–3/cage (pregnant dams), in standard transparent M3 Makrolon® cages lined with bedding material (Scanbur's Aspen wood Bedding, Scanbur, Sweden) in a light/temperature/humidity-controlled environment: 12-h light–dark cycle (light on at 06:00 h), temperature 22°C and 40–50% humidity. Tap water and food pellet (R34 pellet or CBZ diet, Lantmännen, Sweden) was provided ad libitum.

#### Exposure to CBZ

Starting one week before a male mouse was placed in the cage, the female mice were given CBZ diet (3.5 g CBZ/kg in normal R34 pellet, Lantmännen, Sweden) up to the time of delivery. CBZ diet was replaced with normal R34 pellets, without CBZ, directly after delivery. This CBZ diet has been shown to result in serum levels of CBZ in mice well above the minimal effective dose in humans, but below the optimal therapeutic range for human antiepileptic therapy [46]. The control female mice received normal R34 pellet, (Lantmännen, Sweden) during the same time period. The body weight of the newborn pups was not recorded to avoid disturbing the dam and her pups. For the same reasons the pregnant dams were not monitored for signs of toxicity. However, the numbers of litters and the number of survived pups were noted when the mice were three weeks old. The body weight was measured at five weeks of age.

Female and male BALB/c offspring were used to investigate the effect of prenatal CBZ exposure on the hippocampal and cortical neuronal cell counts in newborn (n = 15) and in young (five weeks old, n = 15) mice. Another group of 5 weeks old male and female BALB/c mice was used to investigate the effect of prenatal CBZ exposure on the cognitive abilities in the PA test (n = 19). Two separate groups of female and male C57BL/6 offspring were used to investigate the effect of prenatal CBZ exposure on spatial learning and memory in the Morris water maze test (age: five weeks (n = 11) and nine weeks (n = 10)). The C57BL/6 mice were used, since BALB/c mice were poor performers in this test.

### Perfusion

Newborn pups were decapitated and the brains dissected and incubated in fixative solution (4% paraformaldehyde and 0.4% picric acid in 0.16 M PBS, pH 7.4) for 2 h at room temperature (RT). The brains were then rinsed and stored in sucrose (10% in PBS, 0.1% sodium azide) at 4°C and thereafter snap-frozen in dry-ice-cooled iso-pentane.

5 weeks old mice were deeply anesthetized by pentobarbital and then perfused intracardially at forced pressure with 10 ml Ca^2^-free Tyrode's solution including 0.1 ml heparin, followed by 50 ml of the fixative solution. The brains were dissected and postfixed in the same fixative for 1 h at RT, rinsed and stored in the sucrose solution at 4°C, and thereafter snap-frozen.

### Immunohistochemistry

The brains were cryosectioned in 30 µm coronal sections encompassing the entire hippocampus in a cryostat and mounted on Super-Frost slides. Mouse on mouse (M.O.M) kit for immunodetection (Vector, Burlingame, CA) was used for NeuN staining according to the manufacturer's protocol. The sections were incubated with the primary antibody (anti-NeuN, 1∶100, Chemicon) for 24 h at 4°C and with biotinylated secondary antibody (anti-mouse IgG, 1∶250, M.O.M kit) for 1 h in RT. To enhance signals, ABC Vectastain Elite Reagent (Vector) was applied for 40 min at RT. After detection with 3′3-diaminobenzidine substrate (DAB, Sigma) for 30 seconds in RT the slides were dehydrated and mounted with Pertex (Histolab, Gothenburg Sweden).

### Stereology

The “Optical fractionator” [47], [48] was used to estimate the number of neurons in the dentate gyrus (DG), in the CA1 and CA3 regions of the hippocampus, in cortex cerebri and in amygdala of CBZ exposed and control mice. In newborn mice, neuronal cell counts were determined in the rostral part of hippocampus (−3.5 mm from Bregma to −4.5 mm from Bregma) [49] combining the hippocampal regions CA1 and CA3. Cell counts in five weeks old mice were determined through the entire hippocampus (−0.9 mm from Bregma to −3.9 mm from Bregma) [50]. Here, neurons in DG were counted as one entity whereas neuron counts from the CA1 and CA3 regions were combined. Neuronal cell counts in cortex cerebri and amygdala (−1.6 mm from Bregma to −2.1 mm from Bregma) were also counted [50].

Every tenth section was systematically sampled after that the first section within the first interval had been chosen randomly. An unbiased counting frame of known area was superimposed on the field of view by stereological analysis software (Stereologer™, SPA inc., VA, USA). These counting frames were then automatically and systematically distributed throughout the marked region originating from a random starting point. The optical fractionator estimates are unbiased, since no assumptions are made about cellular shape and size, and not affected by tissue shrinkage during tissue processing. Before counting an area of interest, the areas were manually outlined using a 10× lens; whereas a 60× lens with a numerical aperture of 1.4 was used for cell counts. Only cells that fell within the frame or bordered the inclusion lines were counted, while cells touching exclusion lines were omitted. These steps were performed with a light microscope (Olympus BH-2) modified for stereology with the following components: a computer-driven motorized Märzhäuser stage (Märzhäuser Wetzlat GmbH & Co. KG, Wetzlar-Steindorf, Germany), a microcator (Heidenhain, Traunreut, Germany) and a CCD camera. All stereological analyses were conducted in one hemisphere of the brain.

### Passive Avoidance

The PA task is an associative learning paradigm based on contextual fear conditioning (Pavlovian conditioning), involving neuronal circuits in the limbic forebrain, such as hippocampus and amygdala [44], [51]. In the step-through PA procedure, performed in a two-compartment box, the suppression of the innate preference of rodents for the dark compartment following the exposure to an inescapable foot shock is defined as PA behavior [44], [45], [52], [53]. Memory retention was tested in a computer-controlled PA (TSE-Systems GmbH, Homburg, Germany).

The experiments were performed between 8 a.m. and 3 p.m. Test of memory retention was performed 24 hours after the training [52], [53]. During training the mouse was placed in a bright compartment (BC, 330 lux) (280×155×160 mm) for 60 s. Then the door between the compartments opens and the mouse has free access to the dark compartment (DC) (280×155×160 mm). Upon entering the DC the door closes after 3 s and the mouse received weak electrical current (US) (duration 1 s) 0.30 mA. The mouse was left in the dark compartment for 60 s after the aversive cue (US) had been presented to increase the association of the context and the US [52], [53]. In the retention test the mouse was placed in the BC with the door closed. After 15 s the door was opened and the mouse had free access to both compartments for 10 min, 600 s. Memory retention was examined by measuring the latency time to the first transfer from the BC to the DC with a cut-off latency of 10 min (600 s) [45], [52], [53]. After the end of each test, the arena was cleaned and deodorised after each animal using 70% ethanol.

### Morris Water Maze

The water maze (180 cm in diameter; 45 cm in height) was filled to a depth of 28 cm with tap water (23°C) and placed in the center of a room surrounded by several cues. All studies were performed under dim light conditions (Ögren et al., 1996). Pre-training was performed during three days followed by training during six days, both with four 90 s long trials/day. A 60 s long retention test was performed twenty-four hours after the last training session.

During pre-training the platform (10 cm in diameter) was located 1 cm below the water surface, but was made visible for the animals. The platform location was changed every trial and the periphery of the water tank was covered by a curtain to prevent access to extra-maze cues. During training the platform was located 1 cm below the water surface and invisible to the animal. The platform location remained constant over the six days of training, but was removed at retention on day seven. Before starting the experiments the mice were allowed to habituate to the water maze room for one hour. The water maze system (Water Maze Software, Edinburgh, UK) allow measurements of latency to find the platform, swim distance, swim speed, thigmotaxic behavior as well as quadrant and zone analyses (Cain et al., 1996; Steele and Morris, 1999; Luttgen et al., 2005). A circular area (radius 20 cm from the center of the platform) was defined as the target zone, equivalent to 4.9% of the total water maze area. Thigmotaxic swimming, was defined as the percentage of total time the mice spent swimming within 10 cm from the walls of the water maze.

### Statistical analysis

The results are presented as means ±SEM. The data were analyzed for normality. Normally distributed data were analyzed with parametric tests. Differences between groups were tested with one way-analysis of variance (ANOVA) in the PA task. Student's t test was applied to analyse cell counting data from both new-born and five-week old animals.

The behavioural data were first visually assessed for normality by the sample distribution. Normally distributed results were analysed with parametric test. Differences between the groups were tested with one way- analysis of variance (ANOVA). Significant differences between groups were tested with the Fisher LSD test. The nonparametric test Kruskall Wallis was used for data that were not normally distributed. *P*<0.05 was regarded as statistically significant. All data were analyzed with the computer software program Statistica® 7.0.

## Results

### Prenatal exposure of carbamazepine reduced the number of hippocampal pyramidal neurons in newborn BALB/c mice with 50%

**Figure 1:**
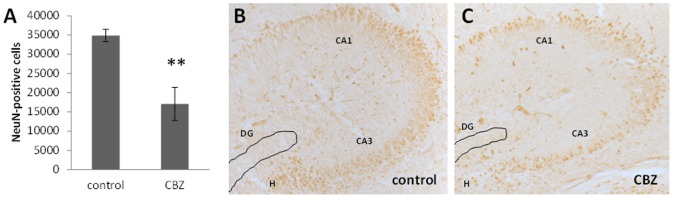
The effect from *in utero* exposure to CBZ on newborn mice. A) Prenatal exposure of CBZ reduces the number of mature pyramidal neurons in hippocampal CA1 and CA3 regions in the newborn mouse. A newborn control mouse hippocampus (B) and a CBZ treated mouse (C). The brown cells (NeuN-immunoreactive cells), which are indicative of mature neurons are more frequently occurring in the non-treated animals (B). There are few NeuN-immunoreactive cells in the newborn mouse dentate gyrus (DG), in agreement with that granule cells mature at later developmental stages compared to pyramidal cells in the CA1 and CA3 regions and in the hilus (H) region. The analysis is performed in the dorsal hippocampus. Values are mean ±SEM. n = 8 controls, n = 7 CBZ treated animals, ** *P* = 0.0013. Scale bar: B) 100 µm C) 25 µm.

Prenatal CBZ exposure reduced the number of mature neurons (NeuN-immunoreactive cells) in the CA1 and CA3 regions of hippocampus (*P* = 0.0013, Fig. 1A) by 50% compared to the control group not exposed to CBZ. There were no mature neurons in the dentate gyrus in the newborn mice (Fig. 1B).

### Prenatal CBZ exposure had long-term effects on hippocampal and cortical pyramidal cells in young BALB/c mice

**Figure 2:**
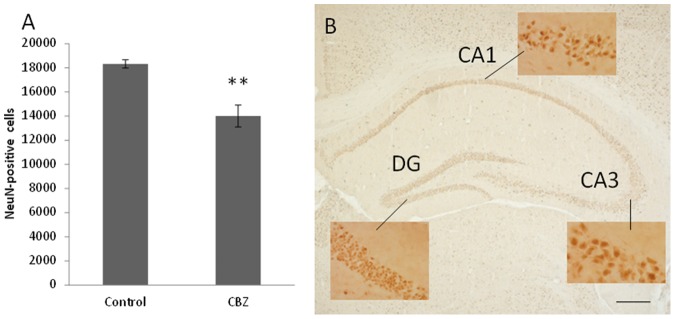
The effect from *in utero* exposure to CBZ on hippocampus in 5 week-old mice. A) Down-regulation of hippocampal CA1 and CA3 pyramidal neurons after prenatal exposure of CBZ are still found in 5 week-old mice. Prenatal CBZ exposure does not affect neurons (granule cells) in the dentate gyrus (DG). B) A representative picture of hippocampus from one brain hemisphere in a five week-old mouse (scale bar 250 µm). High-magnification images illustrate cell morphology of granule cells in DG and pyramidal cells in CA1 and CA3, where the stereological cell-counting were performed. n = 8 controls, n = 7 CBZ treated animals, ** *P* = 0.0004

Prenatal CBZ exposure reduced the number of mature neurons in hippocampal CA1 and CA3 regions in 5 week-old mice, compared to non-exposed mice (*P* = 0.0004, Fig. 2A). CBZ exposure had no effect on neurons in the dentate gyrus (data not shown).

**Figure 3:**
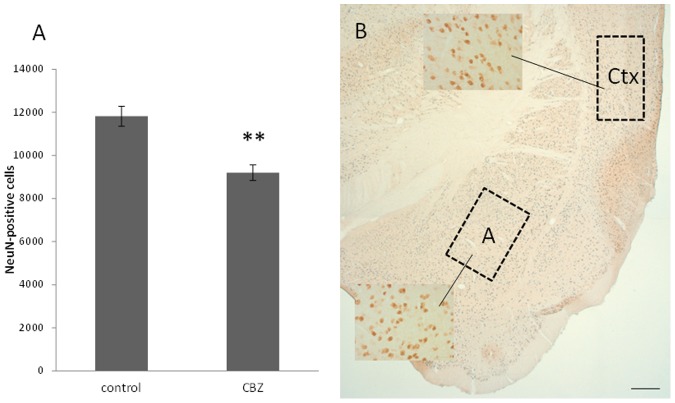
The effect from *in utero* exposure to CBZ is detected in different parts of the brain. A) Prenatal exposure of CBZ decreases the number of pyramidal cells in cortex cerebri of five-week old mice. CBZ did not alter neuronal number in amygdala. B) Representative picture of one brain hemisphere in a five-week old mouse at the level where stereological counting was performed in cortex cerebri (Ctx) and amygdala (A). High-magnification images illustrate cell morphology of pyramidal cells in cortex cerebri and spiny neurons in amygdala (scale bar 250 µm). n = 8 controls, n = 7 CBZ treated animals, ** *P* = 0.0008

Stereological counting of NeuN-positive neurons in cortex cerebri revealed that the number of mature neurons was lower in mice that had been exposed to CBZ *in utero* than in non-exposed mice, (*P* = 0.0008, Fig. 3A). However, CBZ did not affect neurons in amygdala.

### Prenatal CBZ exposure had no effect on learning and memory in young mice

**Figure 4:**
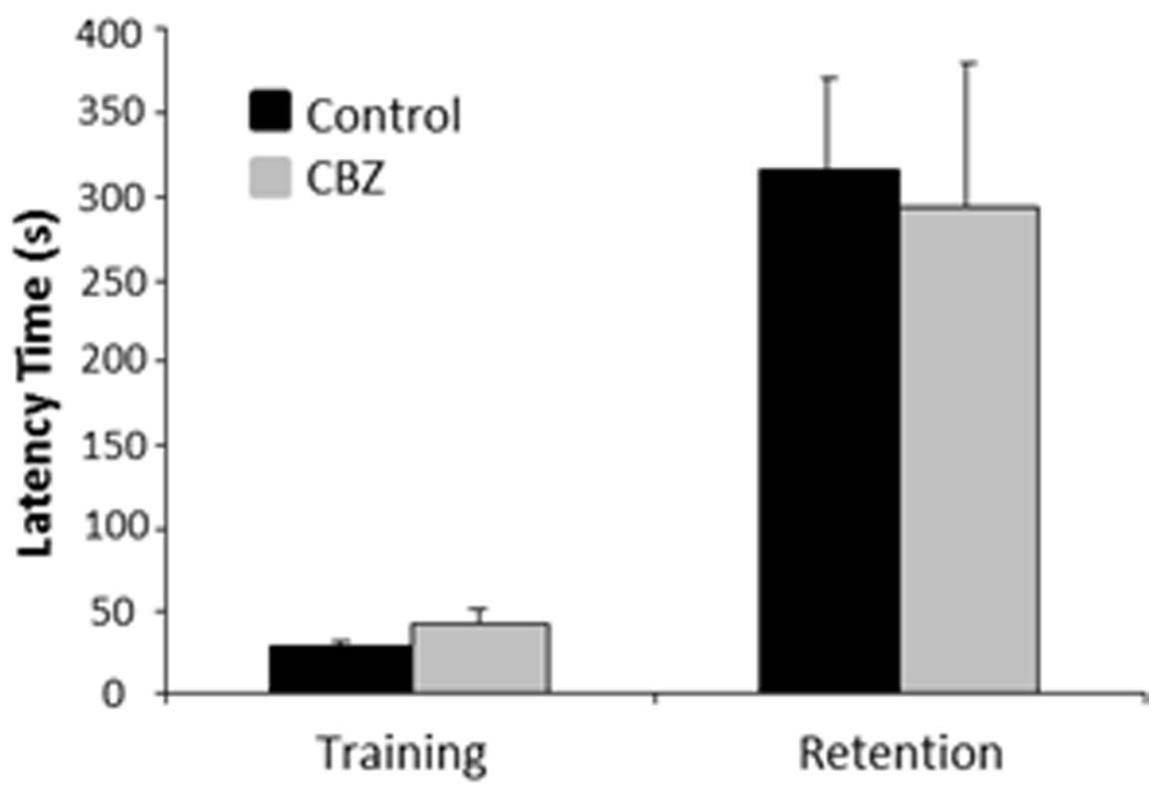
*In utero* exposure to CBZ did not alter emotional memory in 5 week-old mice investigated in the passive avoidance test. There was no effect on training latency or retention latency by prenatal CBZ-exposure. The black bars indicate non-exposed mice (n = 13) whereas the grey bars indicate CBZ-exposed mice (n = 6). Bars indicate mean values and error bars indicate ±SEM.

**Figure 5:**
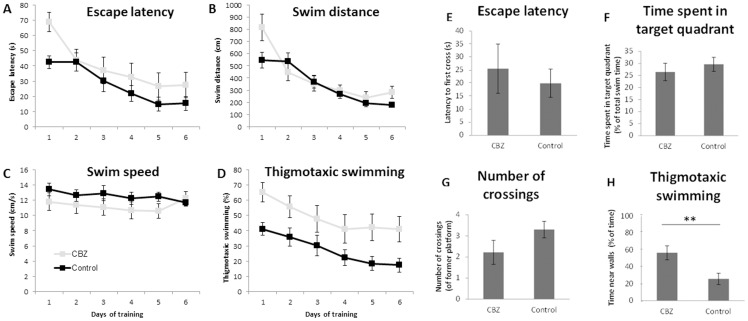
The effects of prenatal CBZ exposure on learning and memory investigated in the Morris water maze test. Figure panels 5 A–D describe the six training days and panels 5 E–H the retention test. *Training sessions*. 5A) There was no significant difference, but a trend, that CBZ-exposed mice were impaired in their ability to improve their escape latency to the hidden platform by training (*P* = 0.098). B) The CBZ-exposed mice had a significantly different swimming distance pattern over training compared to the control mice (*P* = 0.025). After training number 2, the CBZ-exposed mice reduced their swimming distance less by training than the control mice did.C) There was no significant difference in change of swimming speed over time between the CBZ-exposed mice and the controls during training (*P* = 0.39). D) Although there was no effect of training on the time the mice in both groups swam near the walls of the pool (thigmotaxic swimming) (*P* = 0.44), the CBZ-exposed mice tended to have higher thigmotaxic swimming all the days of training (*P* = 0.073). *Retention*. 5E) There was no significant difference in time for the CBZ-exposed and the control mice to find the former platform position, escape latency (*P* = 0.44). F) The two groups of mice spent the same proportion of total swim time in the target quadrant, where the former platform position was (*P* = 0.38). G) The number of times crossing the platform position did not differ between CBZ-exposed and control mice (*P* = 0.79). H) Thigmotaxic swimming; CBZ-exposed mice swam significantly longer time near the walls of the pool during retention (*P* = 0.0092) compared to controls, suggesting that they were more stressed. Bars indicate mean values and error bars indicate ±SEM. ** P<0.01, n = 10 CBZ-exposed mice, n = 11 control mice.

The step-through latency retention in the passive avoidance test was similar between CBZ-exposed and non-exposed BALB/c mice demonstrating that prenatal treatment with CBZ had no effect on amygdala-hippocampal-dependent emotional type of learning and memory (Fig. 4). Likewise, the results from the Morris water maze test showed that prenatal CBZ exposure had no effect on hippocampal-dependent spatial learning and memory (Fig. 5). However, the CBZ exposed C57BL/6 mice displayed an anxious and thigmotaxic behaviour demonstrated by that they spent more time closely to the walls of the pool compared to the non-exposed mice (*P* = 0.0092, Fig. 5D, H).

### Prenatal treatment with CBZ decreased the number of litters, weaned pups and body weight

The number of litters/dam was reduced two times as a result of CBZ exposure during pregnancy. Non-exposed dams had 4 litters/dam during the breeding period while the CBZ exposed dams only had 2 litters/dam. The average number of weaned pups/dam and litter was lower in the group prenatally exposed to CBZ (2.8 pups/dam and litter) compared to non-exposed dams (7.5 pups/dam and litter). In addition, five weeks old mice prenatally exposed to CBZ had a significantly lower body weight than non-exposed mice (*P* = 0.001, Table 1).

**Table 1:** Prenatal exposure to carbamazepine (CBZ) influences mouse body weight. Exposed mice had a lower body weight than non-exposed mice.

	BALB/c [5 weeks]
**CBZ-exposed mice**	14.7±0.48 (*6*)***
**Non-exposed mice**	17.7±0.30 (*13*)

The weight is presented in grams and as mean ±SEM (*n*).

***P<0.001 compared to non-exposed mice.

## Discussion

This is the first study, to our knowledge, investigating the effects of prenatal exposure of CBZ on hippocampal, amygdala and cortical neurons, which are indeed important areas for learning and memory. The major findings in this study are that prenatal exposure of CBZ reduces the number of neurons in the new-born mouse hippocampus, indicated by a 50% reduction of NeuN-immunoreactive cells and that prenatal exposure to CBZ also affects the cell population in five-week old mouse hippocampus and cortex cerebri. Thus, the neurodegenerative effects from prenatal CBZ exposure is long-lasting and are not restricted to hippocampus but also found in cortex cerebri. Interestingly, reduction of neurons were found in hippocampal CA1 and CA3 and cortex cerebri pyramidal cells but not in granule cells in the dentate gyrus and medium spiny neurons in the amygdala. Some cell types may be more sensitive to teratogens than others, in agreement with for example Charriaut-Marlangue et al., Crepel et al and Pellegrini-Giampietro et al. [54], [55], [56]. Since granule cells primarily develop during the early postnatal period, which is after CBZ exposure, their development may have been unaffected [57], [58]. In addition, Cochrane (2009) suggests that AED exposure *in utero* can achieve a precise type of cognitive disturbance by generating a more specific or strong effect in certain brain areas compared to others [9].

Based on the inconclusive results in clinical studies of the effect of prenatal CBZ exposure on cognition [17], [20], [21], [29], [38], [39], [40], [41], [42], [43] we examined the effect of prenatal CBZ exposure on learning and memory in young (5 week-old) BALB/c mice using the Passive Avoidance (PA) test which involves neuronal circuits in the limbic forebrain, such as hippocampus and amygdala. Our intention was, in addition to the PA test, to also investigate BALB/c mice in the Morris water maze task, which is mainly a hippocampus-dependent spatial learning and memory task [59], [60]. However, the BALB/c mice displayed an abnormally high amount of thigmotaxic behaviour, in which they swam around the walls of the pool almost continuously, without any attempt to reach the platform and to get out of the pool. The behaviour could be viewed as a displacement behaviour, displayed in place of other behaviours, when neither being in the water nor out in the open was enough rewarding to perform. The desire to avoid the open of the pool mirrors a higher level of emotionality in this mouse strain. The BALB/c is considered to be a more anxious mouse strain than the C57BL/6 [61], [62]. Since the escape latency in BALB/c could be due to both anxiety and cognition we evaluated the effect of prenatal CBZ exposure on spatial cognition using the C57BL/6 in the Morris water maze. Prenatal CBZ treatment had no detectable effects on learning and memory, neither in the PA nor in the Morris water maze test. These results are in line with most of the clinical investigations performed, which demonstrate that CBZ has minor effects on cognition, especially compared to VPA [17], [20], [42], [43].

Prenatally CBZ treated C57BL/6 mice also displayed the anxiety-like behaviour, thigmotaxis, in the Morris water maze. Whether this is due to a direct CBZ toxic effect on the neural circuits involved in regulating anxiety or an overall effect of CBZ on homeostasis is unknown. It could also be due to fewer cage mates (or occasionally none) for the CBZ exposed animals. The decreased body weight found in the prenatally treated mice could be associated with the increased anxiety level in these animals. Decreased body weight and increased level of anxiety could also separately be a result of CBZ toxicity mentioned in the below section. Whether CBZ has a general toxic effect on the brain neuronal network, or if it hits specific networks, is a question for further investigations.

Pregnant dams treated with CBZ got two times fewer litters/dam compared to non-exposed dams. The average number of weaned pups/dam and litter was also lower in the group prenatally exposed to CBZ compared to non-exposed dams recorded at an age of 3 weeks. In addition, prenatal CBZ exposure also decreased the body weight recorded at five-weeks of age. The decreased number of weaned pups prenatally exposed to CBZ could be due to impaired mating, impaired survival of embryos, absorption of foetuses, less nutrition during the gestation period, complicated parturition, impaired maternal care as well as impaired feeding behaviour or success in competition for food. The role of CBZ in different stages of development as well as on the maternal behaviour and pup response needs to be further elucidated.

CBZ has been demonstrated to induce mitotic arrest in human kidney-derived cell lines [63]. Postnatal CBZ administration to rats on day 7/day 8 did not result in detectable effects on cell proliferation, neurogenesis or cell death [35], [36]. The effects of CBZ exposure may be different depending on if the exposure is postnatal or prenatal. The reduction of neurons found in this investigation could possibly be caused by a decrease in cell proliferation, altered neurogenesis or neurodegeneration. AEDs can down-regulate neurotrophic factors, such as BDNF and NT-3 [34]. It is thus possible that CBZ down-regulated the trophic support for neurons and that this in turn caused neuronal death. AEDs have also been demonstrated to alter intracellular pathways involved in survival promoting signalling. Further studies are needed to address possible mechanisms behind the decrease of the neuronal cell population found after prenatal CBZ exposure.

This is the first study to investigate prenatal CBZ treatment and the effects on brain neuronal cell populations in combination with learning and memory. However, there are some limitations in this study. 1. As already mentioned, BALB/c could be used only in one of the learning and memory tests performed. However, similar to the lack of detectable effect of prenatal CBZ exposure on emotional learning in BALB/c, there was no effect on spatial learning in the C57BL/6 mice. 2. Toxicity of CBZ in pregnant mice or CBZ effects on new-borns, were not investigated in this study. Body weight was only recorded in five-week old mice. The CBZ dose used has been shown to result in serum levels of CBZ well above the minimal effective dose in humans, but below the optimal therapeutic range for human antiepileptic therapy [46]. 3. The CBZ-exposed mice were fewer than those not exposed to CBZ in the passive-avoidance test. Still, adequate statistical models and analyses were performed.

The last Cochrane review (2009) stated the necessity to generate data establishing the effects of *in utero* AED exposure on child development and cognition [9]. When translating the findings of our study to humans the differences in brain developmental stages between rodents and humans should be considered, where the human brain goes through more stages *in utero* than the rodent brain does [64], [65] and also the longer drug-exposure time *in utero* for human fetuses compared to rodent fetuses.

In conclusion, the present study demonstrates that *in utero* exposure to CBZ produces a drastic reduction of mature hippocampal and cortical neurons. Interestingly, no apparent cognitive impairment was detected in the PA test or in the Morris water maze despite this huge reduction of neuron count. This proposes that the loss of neurons in brain areas important for cognition possibly can be compensated for by long-term structural rearrangements of neuronal networks. However, another interpretation may be that CBZ does not affect emotional memory and spatial learning, but rather influence other aspects of cognition like intellectual ability and social interaction. Further studies are required to scrutinize the influence of the CBZ-caused loss of neurons on cognitive functions.
